# Metabolic syndrome in type 2 diabetes: comparative prevalence according to two sets of diagnostic criteria in sub-Saharan Africans

**DOI:** 10.1186/1758-5996-4-22

**Published:** 2012-05-31

**Authors:** Andre P Kengne, Serge N Limen, Eugene Sobngwi, Cathérine FT Djouogo, Christophe Nouedoui

**Affiliations:** 1 NCRP for Cardiovascular and metabolic diseases, South African Medical research Council & University of Cape Town, Cape Town, South Africa; 2 Higher Institute of Health Sciences, Bangangte, Cameroon; 3 Yaounde Central Hospital and Faculty of medicine and biomedical sciences university of Yaounde 1-Cameroon, Yaounde, Cameroon; 4 Institute of Health and Society; The Medical School, University of Newcastle Upon Tyne, Newcastle, UK; 5 General Hospital Yaounde and Faculty of medicine and biomedical sciences university of Yaounde 1-Cameroon, Yaounde, Cameroon

**Keywords:** Metabolic syndrome, Diabetes mellitus, Prevalence, Concordance, Cameroon, Sub-Saharan Africa

## Abstract

**Background:**

Available definition criteria for metabolic syndrome (MS) have similarities and inconsistencies. The aim of this study was to determine the prevalence of MS in a group of Cameroonians with type 2 diabetes, according to the International Diabetes Federation (IDF) and the National Cholesterol Education Programme Adult Treatment Panel III (NCEP-ATP III) criteria, and to assess the concordance between both criteria, and the implications of combining them.

**Methods:**

We collected clinical and biochemical data for 308 patients with type 2 diabetes (men 157) at the National Obesity Center of the Yaounde Central Hospital, Cameroon. Concordance was assessed with the use of the Kappa statistic.

**Results:**

Mean age (standard deviation) was 55.8 (10.5) years and the median duration of diagnosed diabetes (25^th^–75^th^ percentiles) was 3 years (0.5–5.0), similarly among men and women. The prevalence of MS was 71.7% according to the IDF criteria and 60.4% according to NCEP-ATP III criteria. The prevalence was significantly higher in women than in men independently of the criteria used (both *p* < 0.001). Overall concordance between both definitions was low to average 0.51 (95% confidence interval: 0.41–0.61). Combining the two sets of criteria marginally improved the yield beyond that provided by the IDF criteria alone in men, but not in the overall population and in women.

**Conclusions:**

The IDF and NCEP-ATP III criteria do not always diagnose the same group of diabetic individuals with MS and combining them merely increases the yield beyond that provided by the IDF definition alone. This study highlights the importance of having a single unifying definition for MS in our setting.

## Background

Metabolic syndrome (MS) is a group of clinical and biological abnormalities that confers a greater risk of type 2 diabetes, cardiovascular (CVD) [1] and liver diseases [2]. The different components of MS were initially described by Reaven in 1988 under the appellation of “syndrome X” [3]. These include abdominal obesity, higher-than-optimal blood pressure, disorders of glucose metabolism and abnormal lipid profile [4]. Although still debated, the underlying feature of all these abnormalities seems to be insulin resistance [5]. Regardless of the presence of any abnormalities of glucose metabolism, individuals with MS are at increased risk of type 2 diabetes [6]. The co-occurrence of diabetes mellitus and MS potentiates the cardiovascular risk associated with each of the two conditions. Characterizing MS in the presence of diabetes is therefore beneficial for the purpose of cardiovascular prevention. However, instruments for diagnosing MS are likely to vary substantially.

Over the last decade, several sets of criteria have been suggested for the diagnosis of MS. These include the criteria by the World Health Organization (WHO, 1998) [7], the European Group for study on insulin Resistance (EGIR) in 1999 [8], the National Cholesterol Education Program Adult Treatment Panel III (NCEP-ATP III) in 2001 [9] and the International Diabetes Federation (IDF) in 2005 [4]. These criteria share common ground in the sense that they all acknowledge disorders of glucose metabolism, hypertension, dyslipidaemia and obesity as components of metabolic syndrome. They also have areas of inconsistencies, particularly regarding the threshold levels for defining the abnormalities and how these should be combined to define metabolic syndrome. The implication of these inconsistencies is that the yield of various criteria may vary significantly in the same population. This can have potentially undesirable consequences for risk stratification and prioritization of patients for preventive treatment. For instance, a patient may be denied such treatment on the basis of one set of criteria, why he would be eligible using a different set of criteria.

We are not aware of studies at the national or regional level that have assessed and compared the output of various criteria for defining MS in people with diabetes in sub-Saharan Africa. The growing population of individuals with diabetes in the region invites reliable tools to backup strategies for improving their cardiovascular health [10]. Accordingly, the aim of this study therefore was to assess the concordance of two sets of definition criteria for MS in a population of individuals with type 2 diabetes in Cameroon.

## Results

### Characteristics of the study population

A total of 308 type 2 diabetic participants were recruited, with nearly equal number of men and women. The mean age (standard deviation: SD) of participants was 55.8 (10.9) years, and the median known duration of diabetes was 3.0 (25^th^–75^th^ percentiles: 0.5–5.50) years, with no significant difference between men and women. The mean levels of systolic and diastolic blood pressures and prevalence of hypertension were also similar between men and women (Table 1). The mean body mass index (BMI) was higher in women than in men (28.8 vs. 27.0 kg/m^2^; *p* < 0.001), while the prevalence of current smokers was higher in men than in women (8.3% vs. 1.3%; *p* = 0.003), (Table 1).

**Table 1:** Characteristics overall and for men and women

**Variables**	**Men (157)**	**Women (151)**	**P-value**	**Total (308)**
Mean age, years (SD)	55.6 (10.1)	56 (10.9)	0.74	55.8 (10.5)
Known duration of diabetes*	3.0 (0.5-5.0)	3.0 (0.5-5.0)	0.46	3.0 (0.5-5.0)
Mean BMI, kg/m^2^ (SD)	27.0 (4.2)	28.8 (5.0)	<0.001	27.9 (4.7)
Mean waist circumference, cm (SD)	95.9 (9.6)	97.4 (11.0)	0.20	96.6 (10.4)
Mean systolic blood pressure, mm Hg (SD)	142.9 (24.5)	142.8 (25.2)	0.95	142.7 (24.8)
Mean diastolic blood pressure, mm Hg (SD)	84.3 (11.9)	83.0 (11.8)	0.37	83.7 (11.9)
Hypertension, n (%)	84 (53.5%)	83 (55%)	0.80	167 (54.2%)
Smoking, n (%)	13 (8.3%)	2 (1.3%)	0.003	15 (4.9%)
Mean total cholesterol, g/L (SD)	1.78 (0.56)	1.80 (0.52)	0.79	1.79 (0.54)
Mean LDL cholesterol, g/L (SD)	1.10 (0.53)	1.12 (0.49)	0.69	1.11 (0.51)
Mean HDL cholesterol, g/L (SD)	0.53 (0.21)	0.58 (0.22)	0.04	0.56 (0.22)
Triglycerides (g/l)*	0.92 (0.65-1.25)	0.84 (0.58-1.26)	0.41	0.88 (0.63-1.26)
Mean fasting glucose, g/L (SD)	1.57 (0.64)	1.48 (0.61)	0.25	1.53 (0.62)
Mean post-prandial glucose, g/l (SD)	1.96 (0.86)	1.78 (0.81)	0.04	1.87 (0.84)
Glucose control treatments				
Oral agents, n (%)	114 (72.6%)	108 (71.5%)	0.83	222 (72.1%)
Insulin, n (%)	32 (20.4%)	46 (30.5%)	0.04	78 (25.3%)
Insulin and Oral agents, n (%)	8 (5.1%)	18 (11.9%)	0.04	26 (8.4%)
Blood pressure lowering medications, n (%)	65 (41.4%)	73 (48.3%)	0.22	138 (44.8%)
Statins use, n (%)	2 (1.3%)	1 (0.7%)	0.58	3 (1%)
Aspirin use, n (%)	2 (1.3%)	2 (1.3%)	> 0.99	4 (1.3%)

* Values are Median (25^th^ – 75^th^ percentiles).

The biological profile of the study population overall and for men and women is summarized in Table 1. HDL cholesterol level was significantly higher in women than in men (*p* = 0.04) while post-prandial glycaemia was significantly higher in men than in women (*p* = 0.04). No significant difference was noted elsewhere.

Oral anti-diabetic medications were been taken by 222 (72.1%) patients; while 78 (25.3%) were on insulin and 26 (8.4%) patients on both. No patient was on diet alone. More women than men were on either insulin alone, or in association with oral agents (both *p* = 0.04). One hundred and thirty eight (44.8%) were on blood pressure lowering treatments, similarly among men and women (*p* = 0.22). Only 1% of participants were on statin or aspirin (Table 1).

### Crude and age adjusted prevalence of metabolic syndrome

According to the IDF criteria 221 (crude prevalence: 71.7%) participants had metabolic syndrome. The corresponding figures for the NCEP-ATP III criteria were 186 (60.4%), Table 2. The prevalence was significantly higher in women than in men independently of the criteria used (both *p* < 0.001). The age-adjusted prevalence of MS was 64.5% (overall), 55.7% (men) and 72.1% (women) according to the IDF criteria. The corresponding figures for the NCEP-ATP III definition were 55.6%, 43.1% and 68.1% respectively.

**Table 2:** Prevalence of the MS by age group and age standardized prevalence

	IDF criteria				NCEP-ATP III criteria			
Age groups	Men	Women	P	Total	Men	Women	P	Total
<45	11 (52%)	9 (60%)	0.65	20 (56%)	9 (43%)	9 (60%)	0.31	18 (50%)
45-54	31 (62%)	47 (89%)	0.002	78 (76%)	24 (48%)	43 (81%)	<0.001	67 (65%)
55-64	31 (54%)	37 (84%)	0.002	68 (67%)	22 (39%)	34 (77%)	<0.001	56 (55%)
> = 65	18 (62%)	37 (95%)	0.001	55 (81%)	12 (41%)	33 (85%)	<0.001	45 (66%)
Total^a^	91 (58%)	130 (86.1%)	<0.001	221 (71.7%)	67 (42.7%)	119 (78.8%)	<0.001	186 (60.4%)
Total^b^	55.7%	72.1%		64.5%	43.1%	68.1%		55.6%
P^c^	0.78	0.02		0.03	0.80	0.28		0.21

a crude prevalence.

b age standardized prevalence.

c p-value for the difference by age group within a subgroup.

There was a significantly increasing trend in the prevalence of MS by increasing age according to the IDF criteria in the overall group (*p* = 0.03), and in women (*p* = 0.02), but not in men (*p* = 0.78) (Table 2). The prevalence of MS was not significantly different across increasing age strata overall (*p* = 0.21), and in men (*p* = 0.80) and women (*p* = 0.28) respectively according to NCEP-ATP III definition. With the exception of the age stratum <45 years, the prevalence of MS within age group was always higher in women than in men using either criteria.

### Agreement between sets of criteria

The overall concordance between both definitions was 0.51 (95% confidence interval: 0.41–0.61) overall, 0.33 (0.19–0.47) in men and 0.66 (0.50–0.82) in women. The concordance statistics varied across age groups in an unpredictable fashion in the overall group and in men and women (Table 3). But the confidence interval about the kappa statistics between age groups always overlapped, suggesting that differences if any, were likely meaningless.

**Table 3:** Agreement between IDF and NCEP-ATP III criteria (Kappa statistic and 95% confidence interval)

Age group	Men	Women	Total
Total	0.33 (0.19-0.47)	0.66 (0.50-0.82)	0.51 (0.41-0.61)
<45 years	0.62 (0.29-0.95)	0.72 (0.36-1)	0.67 (0.42-0.91)
45-54	0.48 (0.25-0.72)	0.71 (0.45-0.97)	0.61 (0.45-0.77)
55-64	0.07 (−0.17 to 0.32)	0.78 (0.55-1)	0.38 (0.20-0.56)
> = 65	0.34 (0.16-0.65)	0.19 (−0.21 to 0.58)	0.41 (0.19-0.64)

### Cross classification in study population

**Figure 1:**
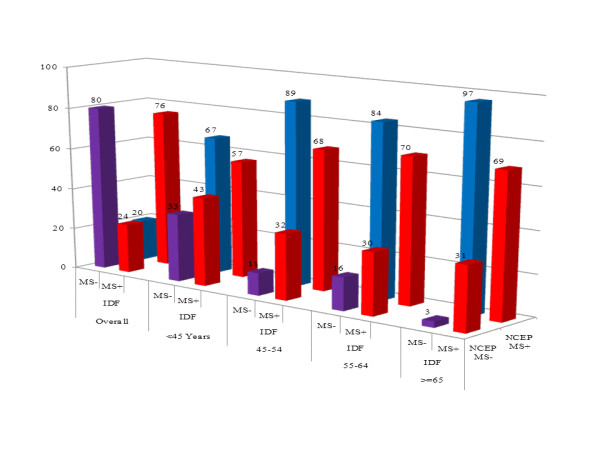
Cross classification of the overall population according to the two sets of criteria. MS- Absence of the metabolic syndrome; MS + Presence of the metabolic syndrome.

Crossed classification of participants based on the two sets of criteria is depicted in Figure 1. At the level of the overall population, 80% of participants classified as not having MS by the IDF criteria were also classified within this category by the NCEP-ATP III criteria, while NCEP-ATP III reclassified the 20% remaining as having MS. Among those diagnosed with MS on the basis of the IDF criteria, the NCEP-ATP III criteria confirmed the diagnosis of MS in 76% and reclassified 24% as not having MS (Figure 1). Among men classified as not having MS using the IDF definition, 23% were reclassified as having MS, while among those diagnosed with MS on the basis of the IDF criteria, 43% were reclassified as not having the syndrome by the NCEP-ATP III criteria. In women 10% of participants without MS based on the IDF definition were reclassified as having the syndrome using the NCEP-ATP III definition, while 10% with the syndrome were further reclassified as not having the syndrome by the NCEP-ATP III definition. Nearly similar pattern of reclassification was observed across age strata in women. In the overall population and in men however, this pattern varied substantially.

### Added value of combining sets of criteria

**Figure 2:**
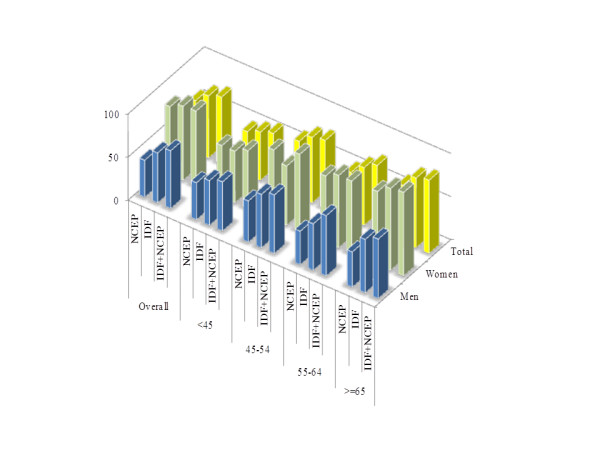
Added value of combining the criteria overall and by age groups.

While the prevalence according to the NCEP-ATP III definition was almost always lower than that according to the IDF definition, combining the two sets of criteria marginally improved the yield beyond that provided by the IDF criteria alone in men (Figure 2), but not in the overall population and in women. In some age subgroups in women, the yield of the NCEP-ATP III criteria was higher than that for the IDF criteria, but their combination was not better than the NCEP-ATP III criteria alone (Figure 2).

## Discussion

Our results revealed high prevalence rates for the metabolic syndrome using both diagnostic criteria but much higher with IDF criteria. These rates were significantly higher in women than in men, independently of the criteria used. Concordance between both definitions was low-to-average overall, and by gender, with the best agreement observed in women. Eighty percents of participants classified as not having MS by the IDF criteria were also classified within this category by the NCEP-ATP III criteria, while NCEP-ATP III reclassified the 20% remaining as having MS. Among those diagnosed with MS on the basis of the IDF criteria, the NCEP-ATP III criteria confirmed the diagnosis of MS in 76% and reclassified 24% as not having MS. Combining the two sets of criteria marginally improved the yield beyond that provided by the IDF criteria alone in men, but not in the overall population and in women.

Few prevalence studies of MS have been conducted in Africa, including in patients with diabetes [11]. Available studies in the general population suggest an increasing prevalence of MS with time, and collectively, have provided variable prevalence of MS ranging from low to as high as figures reported in developed countries [12,13]. Studies in people with diabetes, conducted in large part in Nigeria have been based on different definition criteria and have also provided variables results. One of the earliest was based on a sample of 218 type 2 diabetic outpatients receiving care at the Olabisi Onabanjo University Teaching Hospital in Nigeria from 1999 to 2001 [14]. In this study, Alebiosu and co-workers found a prevalence rate of 25.5%, similarly among men and women using the WHO criteria [14]. This prevalence was half the figure reported subsequently by Adediran et al. [15] in a group of 408 type 2 diabetic individuals at the University Teaching Hospital in Lagos (Nigeria) applying the same definition criteria. The prevalence rate in this study was 51% overall, 44% in men and 56% in women [15]. This was still lower than the 54–59% rate found by Isezuo and Ezunu among diabetic patients at the Usmanu Danfodiyo University Teaching Hospital, Northwestern Nigeria in 2002 [16,17]. More recent studies in the same country have found much higher prevalence rates: 63.6% in one study based on IDF criteria [18], 62.5% in a second based on the NCEP-ATP III criteria [19]; and 25.5% [20], 60% [21], 69% (in study that enrolled only women) [22] and 86% [23] in four studies from the same institution/investigators applying the Joint Interim Statement (JIS) criteria [24]. Elsewhere in Africa, a 43% prevalence rate was reported among 109 diabetic subjects in a tertiary care diabetes clinic in Zimbabwe [25]; and rates of 66.8%, 85.5%, 74% among men and 87.1%, 79.7%, 93% among women, according to the NCEP-ATP III, WHO and IDF criteria, respectively, reported in Seychelles [26]. One study compared the prevalent of MS based on IDF criteria in African and White South African diabetics. Rates of MS in this study were lower in Africans than in Whites (46% vs. 74%) [27].

It follows from the above that prevalence rates found in our study are among the highest so far reported in sub-Saharan Africa. In general however, available reports from Africa have mostly provided non-standardised prevalence rates, which make direct comparison between studies less reliable. Those reports have also been based on patients with variable duration of diabetes, which can further increase the disparities in the prevalence of MS between studies. Indeed, the prevalence of components of MS is likely to increase with increasing duration of diabetes. Our prevalence rates compare with reports from various settings around the world [28-30]. These similarities suggest that the prevalence of the MS in people with type 2 diabetes seems to be independent of ethnic factors as the prevalence rates remain high independent of the definition criteria used.

While some studies in Africa have simultaneously applied different definition criteria for MS in the same population, very few (and perhaps none in people with diabetes) have directly compared the performance of different sets of criteria. One study in the general population in rural South Africa found a good agreement between the JIS criteria on one side and either the IDF (kappa = 0.90) or the NCEP-ATP III (kappa = 0.77) on the other side [31]. This agreement in major ways is expected based on the fact that JIS more or less is just a loose adaptation of the IDF and NCEP-ATP III criteria [24]. Therefore virtually all patients with MS based on either IDF or NCEP-ATP III criteria would also have MS based on JIS criteria. The South African study however did not directly compare the IDF and NCEP-ATP III criteria [21]. In another study in Seychelles, the agreement between the IDF, NCEP-ATP III and WHO criteria was assessed overall and after exclusion of participants with diabetes [14]. The agreement between the IDF and NCEP-ATP III criteria was acceptable-to-good while those for IDF vs. WHO and NCEP-ATP III vs. WHO were only modest-to-acceptable, with in both cases only a modest decrease in the point estimates upon exclusion of participants with diabetes [14]. Both the South African and Seychelles reports were all based on community samples. In our hospital-based sample of treated diabetic individuals with varying duration of the disease, we found a low-to-acceptable agreement between the IDF and NCEP-ATP III criteria. The agreement was particularly poor among men. This is essentially explained by differences in the contribution of abdominal obesity to the definition of MS based on IDF or NCEP-ATP III criteria [4,9].

Though both definitions identified a high prevalence of the metabolic syndrome, combining the two sets of criteria marginally improved the yield beyond that provided by the IDF criteria alone in men, but not in the overall population and in women. This may likely imply that using both definitions is of no value but instead a need to use a single and adaptable definition to a particular population. The JIS criteria published in 2009 aimed to achieve the above [24]. While the JIS Committee has agreed on the principle of a definition with no obligatory component, it also explicitly recognized two facts: 1) the abdominal obesity (waist circumference) component should be based on population-specific threshold; 2) Most patients with type 2 diabetes mellitus will have the metabolic syndrome by the JIS criteria. There is currently no specific cut-off of waist circumference for African Africans. Only one study has attempted to define such a cut-off based on cross-sectional data in South-Africa [31]. This cut-off has not yet been validated for incident outcomes such as cardiovascular disease, and may therefore not be recommended for use in other settings. The study however, suggests that African-specific cut-off would likely be different from the Caucasian cut-off currently recommended for use in Africa [31]. As predicted, studies that have applied the JIS criteria found very high rate of MS among people with diabetes in Africa [23]. All participants in our sample (data not shown) qualified for MS based on the JIS criteria. This suggests that, until the appropriate cut-offs for waist circumference specific to Africans are well defined; the application of the JIS criteria in people with diabetes in this setting would be of limited contribution.

Our study has some limitations that must be accounted for when interpreting our findings. This was a cross-sectional study conducted in a referral health institution. Therefore, whether our findings reflect those from a broader population with diabetes at the community or primary care level is not known. However, virtually all studies on MS in Africa have been conducted in tertiary care institutions, which make our findings comparable with those from other countries in the region. Our study was based on patient files and registers, and therefore we had no control on the quality of data used in the study. It is possible that our estimates are imprecise due to measurement errors. While imprecision in the levels of risk factors could affect the prevalence of MS, it was less likely to affect the agreement statistics between sets of criteria.

## Conclusions

This study despite its limitations contributes to mapping the prevalence of MS worldwide, particularly with regards to people with diabetes in the African region. Our findings emphasize the growing burden of lifestyle-related non-communicable diseases in countries in epidemiological transition including Cameroon (24), consistent with the ongoing epidemic of obesity worldwide. This study to the best of our knowledge is the first in sub-Saharan Africa describing relevant differences in the application of IDF and NCEP–ATP III MS definitions in people with type 2 diabetes and, moreover it opens doors for further research to monitor the impact of the adverse cardio-metabolic profile on future outcomes of the patients. Just like exploring issues around the characterization of MS in people with diabetes in this setting, it is also relevant to explore avenues for improving the adoption of other approaches to risk stratification such as the use of global absolute risk tools in this context [32].

## Methods

### Study setting

This was a hospital-based cross-sectional study, conducted at the Obesity Centre of the Yaounde Central Hospital. This centre is the out-patient section of the endocrinology service of the same health facility. The study setting has been described in details previously [33]. At the time this study was conducted, the centre was staffed with 3 endocrinologists, one general practitioner, one dietician, a nurse educator, a laboratory technician and 3 nurses. A weekly day hospital handled the annual assessment of patients with diabetes at the centre. Activities performed as part of the day hospital include: medical consultation, therapeutic education, nutritional education, electrocardiogram, fundus oculi, biological exams including fasting and postprandial glycaemia, glycated haemoglobin, lipid profile (high density lipoprotein, triglycerides, total cholesterol, calculated low density lipoproteins), urine multistix, urea, creatinine and uric acid levels, microalbuminuria. Low density lipoproteins levels are calculated using Friedewalds formula as follows: LDL-cholesterol = total cholesterol – HDL-cholesterol – (triglycerides÷5) in grams per litre.

### Study population

This included individuals with type 2 diabetes who had participated in day hospital activities during the period of 2006–2008. The study was approved by the administrative authorities of the hospital, acting institutional review board. Data collected was handled in strict respect of confidentiality. Patient identity was kept anonymous. Data collection used a pre-designed data collection form and included: age in years, sex, type and duration of diagnosed diabetes (in years), past medical history of hypertension and dyslipidaemia and treatments. Anthropometric measurements included height (centimetres), weight (kilograms) and waist circumference (centimetres). Systolic (SBP) and diastolic (DBP) blood pressure were recorded in mmHg. Biological data included the lipid profile (triglycerides, high density lipoproteins, total cholesterol, and low density lipoproteins in grams per litres) and blood glucose levels (fasting and postprandial in grams per litre).

### Definitions

Two definitions for the metabolic syndrome were applied (IDF and NCEP-ATP III). The IDF 2005 definition [4] was based on waist circumference ≥ 80 cm (women) ≥ 94 cm (men) and any two or more of any of the following: fasting triglycerides ≥ 1.5 g/L or triglycerides lowering drugs; fasting HDL cholesterol < 0.4 g/L (men), <0.5 g/L (women); SBP ≥ 130 mmHg and/or DBP ≥ 85 mmHg, or blood pressure lowering treatment; fasting plasma glucose ≥ 1 g/l, or previously diagnosed type 2 diabetes. The NCEP-ATP III definition [9] was based on three or more of any of the following: waist circumference > 88 cm (women) 102 cm (men); fasting triglycerides ≥ 1.5 g/L; fasting HDL cholesterol <0.4 g/L (men), <0.5 g/L (women); SBP ≥ 130 mmHg and/or DBF ≥ 85 mmHg; fasting glucose ≥ 1.1 g/L.

### Statistical analysis

The sample size was calculated with the use of the command *sskdlg*[34] of the statistical software STATA®. This calculation was based on an expected kappa statistic of 0.3, a prevalence of metabolic syndrome of 40% by each set of criteria, a minimum envisaged difference of 0.11 between the kappa statistic and its lower (or upper) 95% confidence interval. Based on these criteria, the required sample size was 298 participants. The *sskdlg* command as described in the STATA® manual is based on the asymptotic variance presented by Fleiss, Cohen and Everitt [35,36] and follows the procedure outlined by Cantor [37]. Data were processed and analysed with the use of Excel® 2007 and SAS/STAT® v9.1 for Windows. Results are presented as percentages, means, and medians. Groups comparison used the chi square tests or equivalents for qualitative variables and the *t*-test of Student and Analysis of the Variance (ANOVA) or non-parametric equivalents as appropriate for quantitative variables. The concordance between sets of criteria for defining metabolic syndrome was assessed with the use of the kappa statistic. Unless otherwise indicated, a p-value <0.05 was considered significant.

## Abbreviations

ANOVA: analysis of the variance; CVD: cardiovascular disease; DBP: diastolic blood pressure; EGIR: European Group for study on Insulin resistance; HDL: high density lipoprotein; IDF: International Diabetes Federation; JIS: Joint Interim Statement; MS: metabolic syndrome; NCEP: National Cholesterol Education Program; SBP: systolic blood pressure; SD: standard deviation; WHO: World Health Organization.

## Competing interests

The author declares that they have no competing interests.

## Authors’ contributions

APK conceived the study design, analyzed and interpreted the data, and drafted the manuscript. NSL assembled the data, interpreted the findings and drafted the manuscript. ES conceived the study and critically revised the manuscript. TCFD critically revised the manuscript. CN conceived the study and critically revised the manuscript. All authors approved the final version and submission of the manuscript.
